# Genetic diversity and population structure of Tongcheng pigs in China using whole-genome SNP chip

**DOI:** 10.3389/fgene.2022.910521

**Published:** 2022-08-25

**Authors:** Jiao Yuan, Xiang Zhou, Guoqiang Xu, Sanping Xu, Bang Liu

**Affiliations:** ^1^ Key Laboratory of Agricultural Animal Genetics, Breeding and Reproduction of Ministry of Education, College of Animal Science and Technology, Huazhong Agricultural University, Wuhan, China; ^2^ The Cooperative Innovation Center for Sustainable Pig Production, Wuhan, China; ^3^ The Engineering Technology Research Center of Local Pig Breed Improvement of Hubei Province, Wuhan, China; ^4^ Department of Agricultural and Rural Bureau, Xianning, China

**Keywords:** tongcheng pig, population structure, genetic diversity, runs of homozygosity, SNP chip

## Abstract

Tongcheng (TC) pigs, distinguished by their superior meat quality, are a Chinese indigenous pig breed. Recently, the genetic resources of TC pigs are under tremendous threat due to the introduction of cosmopolitan pig breeds and African swine fever disease. To promote their management and conservation, the present study assessed genetic diversity and population structure of TC pigs using single nucleotide polymorphism (SNP) markers. A total of 26, 999 SNPs were screened from 51, 315 SNPs in 68 TC pigs. The multi-dimensional scaling (MDS) analysis and neighbor-joining tree revealed that all 68 pigs were from a purebred population. The effective population size decreased over time, and it was 96 prior to generation 20. Both linkage disequilibrium (LD) and neutrality test indicated a low selection of TC pigs with average LD value of 0.15 ± 0.23. Genetic diversity results exhibited a minor allele frequency (MAF) of 0.23, observed heterozygosity (H_O_) of 0.32, expected heterozygosity (He) of 0.31, and nucleotide diversity (Pi) of 0.31. All these parameters indicated a remarkably high genetic diversity of TC pigs. Additionally, 184 runs of homozygosity (ROH) segments were detected from the whole genome of TC pigs with an average ROH length of 23.71Mb, ranging from 11.26Mb to 69.02 Mb. The highest ROH coverage was found on chromosome 1 (10.12%), while the lowest was on chromosome 18 (1.49%). The average inbreeding coefficients based on ROH (F_ROH_) was 0.04%. Fourteen ROH islands containing 240 genes were detected on 9 different autosomes. Some of these 240 genes were overlapped with the genes related to biological processes such as immune function, reproduction, muscular development, and fat deposition, including *FFAR2*, *FFAR4*, *MAPK8*, *NPY5R*, *KISS1*, and these genes might be associated with such traits as meat quality and disease resistance in TC pigs. Taken together, population structure and genetic diversity results suggested that the TC pig represented a valuable genetic resource. However, TC pig breed conservation program remains to be further optimized to ensure adequate genetic diversity and avoid inbreeding depression. Our findings provide theoretical basis for formulating management and conservation strategies for TC pigs.

## Introduction

The Tongcheng (TC) pig, an important Chinese genetic resource, is mainly distributed in Tongcheng country (Hubei Province, China). They are distinguished by superior meat quality, moderate intramuscular fat, and intense flavor (Fan et al., 2006). Currently, TC pig attracts considerable attention due to its strong resistance to highly pathogenic Porcine Reproductive and Respiratory Syndrome Virus (PRRSV) infection (Liang et al., 2017). Over past decades, commercial pig breeds have experienced intensive selection and have been imported into China. They dominate the Chinese pig industry, which poses a huge threat to indigenous pigs including TC pigs, thus resulting in the erosion to unique genetic resources (Wang et al., 2015). Additionally, the outbreak of African swine fever disease has placed TC pigs in danger. These severe challenges make the conservation of TC pigs an urgent and critical task.

An important prerequisite for the development and implementation of comprehensive conservation plans is to know the genetic structure of the existing livestock populations in a given region (FAO, 2015). The primary goal of this study is to investigate the population structure and genetic diversity so as to provide better conservation strategies for TC pigs. Numerous studies have shown that multi-dimensional scaling (MDS) and phylogenetic tree analyses are effective methods for the investigation of population structure, that the proportion of polymorphic loci (P_N_), minor allele frequency (MAF), heterozygosity, and nucleotide diversity (Pi) in combination with linkage disequilibrium (LD) and effective population size (Ne) levels are good indicators reflecting the genetic diversity of the population, and that runs of homozygosity (ROH) and ROH island analyses can reveal the inbreeding of the genome (Cai et al., 2020; Xu et al., 2021). Owing to the rapid development of molecular biology in recent years, a variety of markers and DNA sequences of different species can be determined easily at low cost. Since single nucleotide polymorphism (SNP) markers are widely spread in the genome (Peripolli et al., 2017), they have been employed to study population structure and diversity of indigenous pig breeds in some countries such as China (Liu et al., 2020), Poland (Szmatola et al., 2020), Iberia (Saura et al., 2013), Russia, Kazakhstan, Ukraine (Traspov et al., 2016), Denmark (Cai et al., 2020), and South Africa (Hlongwane et al., 2020).

Therefore, to promote effective conservation and sustainable development of TC pigs, the present study characterized 1) population structure, 2) linkage disequilibrium and effective population size, 3) genetic diversity, and 4) runs of homozygosity. An insight into population structure and genetic diversity of TC pigs is crucial for the formulation of rational management strategies and the conservation of the unique traits of TC pigs.

## Materials and methods

### Samples, genomic data, and quality control

From 15 lineages with pedigree records from the Genetic Resource Preservation Farm (Tongcheng country, Hubei, China), we selected the representative 20 boars and 48 breeding sows as study object. Genomic DNA was extracted from ear tissues using a standard phenol/chloroform method (Moore, 1994). The extracted DNA samples were stored at −20 °C for further analysis.

All the individuals were genotyped with the “Zhongxin- I″ Porcine Chip (Beijing Compusen, China), using Infinium XT 96-Sample BeadChips which could provide the highest throughput array format based on the Infinium data, and a total of 51,315 SNPs were identified. The reference genomes of “Zhongxin- I″ Porcine Chip contained 9 populations of Chinese local pigs such as two-end-black pigs, Sutai, Laiwu, Erhualian, and Bamaxiang. Thus, this “Zhongxin- I″ Porcine Chip was selected for this study.

The quality control was conducted by the software Plink (v 1.90) (Purcell et al., 2007) to exclude unreliable genotypes. Briefly, the individuals with call rate (CR)≤95%, minor allele frequency (MAF) ≤0.01and Hardy-Weinberg equilibrium test (HWE) (*p* ≤ 10^–6^) were excluded (Zhao et al., 2018). Furthermore, those genotypes with genotype missing rate>5% were also excluded. After quality control, a total of 26, 999 SNPs (Supplementary Table 1) were screened from 51, 315 SNPs in 68 TC pigs for subsequent analysis.

### Population structure analysis

#### Multi-dimensional scaling

The Plink (v 1.90) (Purcell et al., 2007) and Tassel (v 5.0) (Bradbury et al., 2007) were used for Multi-dimensional scaling (MDS) analysis, and the data were downscaled to three dimensions for population stratification.

#### Neighbor-joining (NJ) tree

TC pigs are currently a finite population, genetic drift and inbreeding are the two main reasons affecting genetic diversity. The degree of genetic drift and the increment in inbreeding is mainly determined by the smaller number of sexes, namely, the boar. So, the phylogenetic tree of 20 boars was constructed. The 20 boars cover all the pedigrees of TC pigs in the preservation farm.

To estimate the genetic distances among TC pigs, all 26, 999 SNPs were used to calculate the average proportion of alleles shared, Dst, using PLINK(Purcell et al., 2007). The definition of Dst is as follows:

$$D_{st}=\frac{IBS_{2}+0.5+IBS_{1}}{N}$$

Where IBS_1_ and IBS_2_ are the numbers of loci that share one or two alleles at one locus. The genetic distance (D) between all pair-wise combinations of individuals was calculated as follows: $1-D_{st}$ .

Neighbor-joining (NJ) trees were conducted by using MEGA 11 (Tamura et al., 2021) based on the matrix of D.

The classification criteria are based on the requirements of the document “Administrative Measures for Livestock and Poultry Genetic Resources Conservation Farms and Gene Banks” issued by the Agriculture Ministry of China, namely, a single breed that meets the breeding standard is supposed to have no relationship within three generations. Assuming that individual X and parents D and E, there are no relationships between parents D and E within three generations, A is the common ancestor of individuals D and E, the inbreeding coefficients were calculated as follows:

$${\text{F}}_{\text{X}}=\sum \left[{\left(\frac{1}{2}\right)}^{n_{1}+n_{2}+1}\left(1+F_{A}\right)\right]$$

And the relationships between parents is calculated as follows:

$$r_{DE}=\frac{\sum \left[{\left(\frac{1}{2}\right)}^{n_{1}+n_{2}}\left(1+F_{A}\right)\right]}{\sqrt{\left(1+F_{D}\right)\left(1+F_{E}\right)}}$$

Where D and E are non-inbred individuals, and thus the value of F_D_ and F_E_ is 0. According to the formula, F_X_ is 0.03125 and $r_{DE}$ is 0.0625. No kinship within 3 generations and the kinship coefficient of less than 0.0625 are the two criteria for boar pedigree division. And the genetic distance (D) between individuals which is also calculated as D = 1-r.

### Analyses of linkage disequilibrium and effective population size

#### Linkage disequilibrium

r^2^ was used to evaluate Linkage disequilibrium (LD) extent of whole genome of TC pigs, and r^2^ referred to the LD extent of each pair of SNPs per chromosome (Hill and Robertson, 1968). The r^2^ of pairwise SNPs was calculated by using the parameters “plink--file--r2 --ld-window 99,999 --ld-window-r2 0 --out” in Plink (v 1.90) (Purcell et al., 2007). The physical distances between SNPs were divided into 100-kb intervals to visualize the decline of LD, and then the average of r^2^ in each interval was estimated. The distribution of LD was plotted by R package ggPlot 2 (https://ggplot2.tidyverse.org/).

#### Effective population size (Ne)

Ne was estimated based on LD value (Corbin et al., 2012), the formula was as follows:

$$Ne_{\left(t\right)}=\frac{1}{\left(4f\left(C_{t}\right)\right)}\left(\frac{1}{E\left[r_{adj}^{2}\text{|}C_{t}\right]}-\alpha \right)$$

Where Ne_(t)_ is the effective population size prior to t generations; C_t_ is the recombination rate prior to t generations; r_adj_^2^ indicates the estimated linkage disequilibrium after sampling bias was adjusted; and α is a constant.

SNP-marker distances between 0 and 1,000 Mb were divided with 30 distance bins (50 kb each). The r^2^ values corresponding to various distances were used to estimate Ne at different time points using SNeP version 1.1 (Barbato et al., 2015). The time span ranged from prior to generation 1,000 to 1 generation.

### Genetic diversity indices

Genetic diversity is the variety of alleles and genotypes present in the group under study (population, species, or group of species). It is easy to understand that genetic diversity is the extent of heritable variation in a population, or Species. In this study, Seven indices were used to estimate TC-genetic diversity, of which P_N_, MAF, Ho, and He were analyzed by software vcftools and Plink (v 1.90) (Purcell et al., 2007). The sequences were aligned by DnaSP v5 (Librado and Rozas, 2009), and Pi (Nei, 1987) were used to assess nucleotide polymorphisms. Neutrality test was conducted to assess the selective neutrality of mutations based on Tajima’s D (Tajima, 1989), Fu and Li’s F*, and Fu and Li’s D* (Fu and Li, 1993).

### Runs of homozygosity (ROH)

#### Measurement of ROH

To determine ROH, the Plink (v 1.90) parameters and thresholds (Purcell et al., 2007) were set as follows: 1) Sliding window was 20 SNPs across the entire genome; 2) Proportion of homozygous overlapping windows was 5%; 3) Minimum length of a ROH was 1Mb; 4) Maximum gap between consecutive homozygous SNPs was 1,000 kb; and 5) Maximal number of SNPs with missing genotypes was 5 within a ROH, and the maximal number of heterozygous genotype was 1 within a ROH. Since the minimum ROH length was set as 1 Mb, no linkage disequilibrium (LD) -based pruning was needed to exclude short and common ROH induced by LD (Purfield et al., 2012).

The genomic ROH distribution was further investigated. Specifically, the mean number of ROHs per individual, the average length of ROH of population, the total number and average length of ROH per chromosome, and the percentage of chromosomes covered by ROH were calculated.

#### Genomic inbreeding coefficients

Genomic inbreeding coefficients per individual were calculated based on runs of homozygosity (F_ROH_) according to the genome autozygotic proportion, as previously reported (McQuillan et al., 2008):

$${\text{F}}_{\text{ROH}}=\frac{\sum {\text{L}}_{\text{ROH}}}{{\text{L}}_{AUTO}}$$

Where $\sum {\text{L}}_{\text{ROH}}$ is the sum of the length of all ROHs detected from an individual, and ${\text{L}}_{\text{AUTO}}$ is the total length of the autosomal genome covered by SNPs included in the array. we excluded sibling effects on F_ROH_ and calculated F_ROH_ for 41 individuals without full-sib families, according to the pedigrees of 68 pigs provided by the preservation farm.

#### ROH islands and candidate genes

The R software was used to statistically analyze the occurrence frequency for which each SNP per individual fell inside a sliding window. Further, the top 1% of all SNPs across all ROH with the highest occurrences were defined as candidate SNPs under directional selection. All the adjacent SNPs within the top 1% were merged to form ROH islands. Our ROH islands were aligned against porcine Sscrofa11.1. The Ensemble Biomart (http://www.biomart. org) online tool was used to search annotated genes within aligned ROH islands. DAVID (http://david.abcc.ncifcrf.gov/) was employed to determine the function of annotated genes.

## Results

### Population structure

**FIGURE 1:**
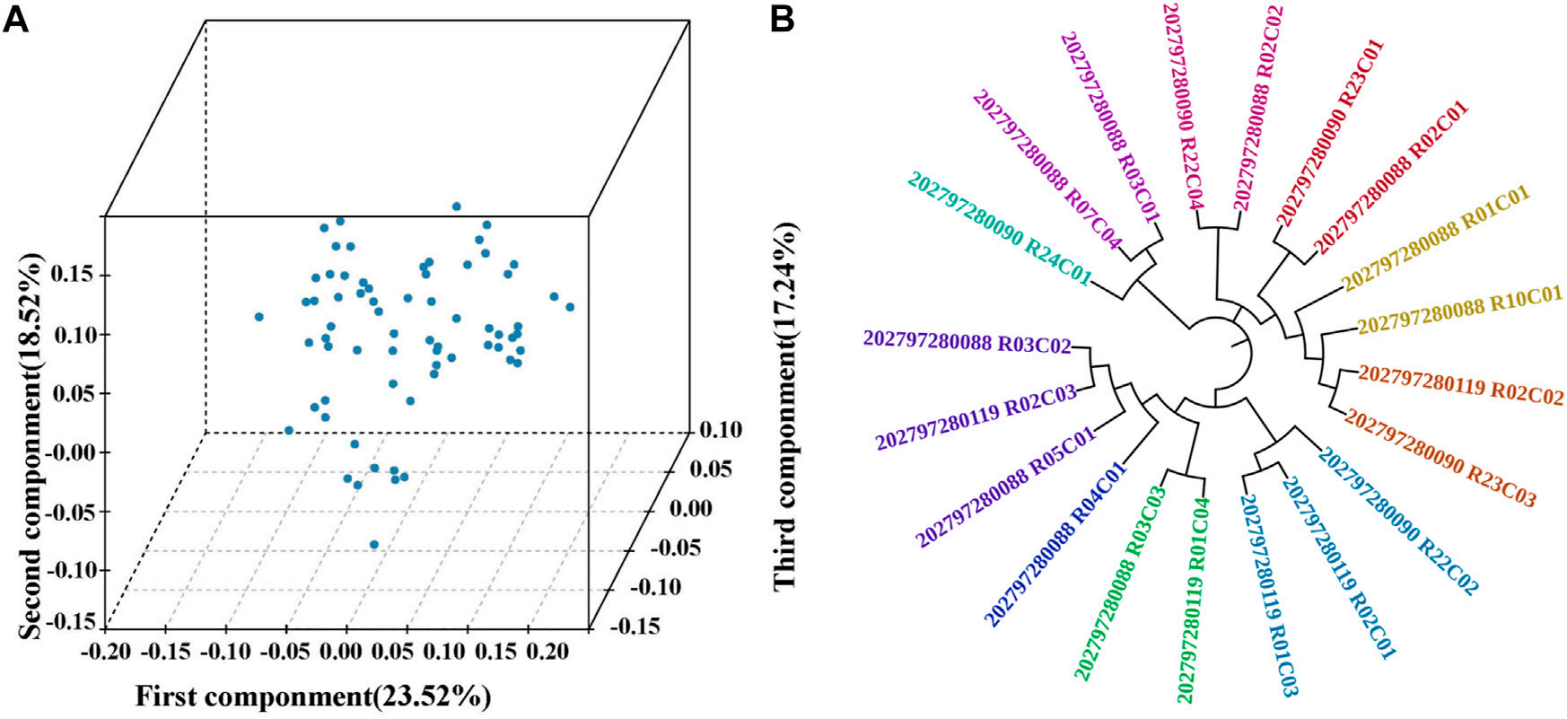
Population structure of Tongcheng (TC) pigs. **(A)** Multi-dimensional scaling analysis of TC pigs. The first three components (first component, second component and third component) were analyzed. **(B)** Phylogenetic relationships (unrooted NJ-tree) between 20 TC boars. 20 boars were divided into 10 families, and individuals with the same color represent one family, while different colors are different families.

MDS analysis was conducted to cluster 68 TC pigs. The eigenvalues of the first three components were 23.52%, 18.52%, and 17.24%, respectively (Figure 1A). All the individuals were closely clustered with no visible separate subgroups. Further, the family structure of 20 boars were reconstructed based on the pairwise genetic distance. And the relationship between families was controlled within 0.0625. The clustering pattern showed that all individuals fell into ten families and the numbers per family ranged from 1 to 3 (Figure 1B), indicating that TC pigs were purebred from various families.

### Linkage disequilibrium and effective population size

**FIGURE 2:**
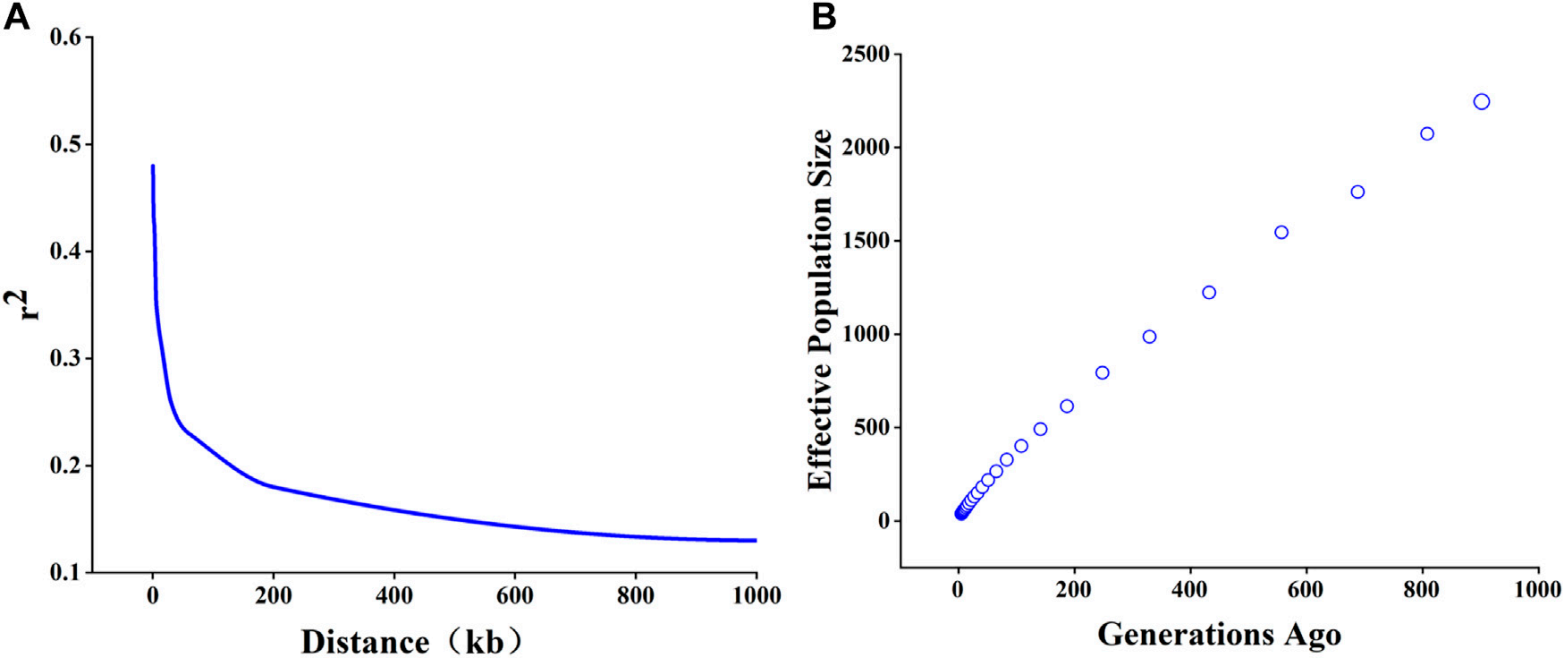
Linkage disequilibrium (LD) decay and effective population size (Ne) of Tongcheng (TC) pigs. **(A)** LD decay of TC pigs. **(B)** Ne of 68 TC pigs. The time span ranged from prior to generation 1,000 to 1 generation.

The mean ± SD of estimated r^2^ were 0.15 ± 0.23. The LD value was decreased with the increase of the distance between SNPs (Figure 2A). When physical distance between adjacent SNPs was 0.5, 1, and 3Mb, the corresponding average r^2^ of TC pigs was 0.48, 0.45, and 0.42. The corresponding physical distance between pair-wise SNPs when r^2^ decayed below 0.3 was chosen as the threshold for the assessment of the extent of LD patterns. Our results indicated that TC pigs had a short LD extent (r_0.3_^2^ = 15 kb). As shown in Figure 2B, Ne exhibited in a sharp decline within the time span from prior to the 1000th generation to the 5th generation. The Ne was approximately 401, 219, 96, 64 prior to generation 100, 50, 20, 12, respectively.

### Genetic diversity

Seven indices were used to estimate genetic diversity of TC pigs, and the results were presented in Table 1. Most TC pigs exhibited a high P_N_, ranging from 0.668 to 0.905. The minor allele frequency (MAF) was 0.23 ± 0.15, varying from 0.01 to 0.50. MAF of more than 23.15% SNPs was higher than 0.40, and that of 22.09% SNPs was lower than 0.10 (Supplementary Figure S1).

**TABLE 1:** Genetic diversity of Tongcheng pigs.

Number of SNPs	Genetic diversity			Nucleotide diversity		
	P_N_	MAF	Ho	He	Pi	
	84.85%	0.23 ± 0.15	0.32 ± 0.16	0.31 ± 0.15	0.31	
	Tajima’s D test		Fu and Li’s D* test		Fu and Li’s F* test	
26, 999	Statistic	Statistical significance	Statistic	Statistical significance	Statistic	Statistical significance
	2.38	*, *p* < 0.05	3.00	**, *p* < 0.02	3.23	**, *p* < 0.02

P_N_, proportion of polymorphic loci; MAF minor allele frequency, Ho observed heterozygosity, He expected heterozygosity, Pi Nucleotide diversity, The following is the same.

In TC pigs, He and Ho ranged from 0.01 to 0.5 and from 0.01 to 0.701, respectively, and Ho (0.32) was slightly higher than He (0.31) (Supplementary Figure S2). The Pi was 0.31. The results of Tajima’s D, and Fu and Li’s F*, and Fu and Li’s D* were mostly positive at the corresponding loci. The means of Tajima’s D test and Fu and Li’s D* test for the entire population was 2.38 (*p* < 0.05) and 3.00 (*p* < 0.02), respectively.

### Runs of homozygosity

**FIGURE 3:**
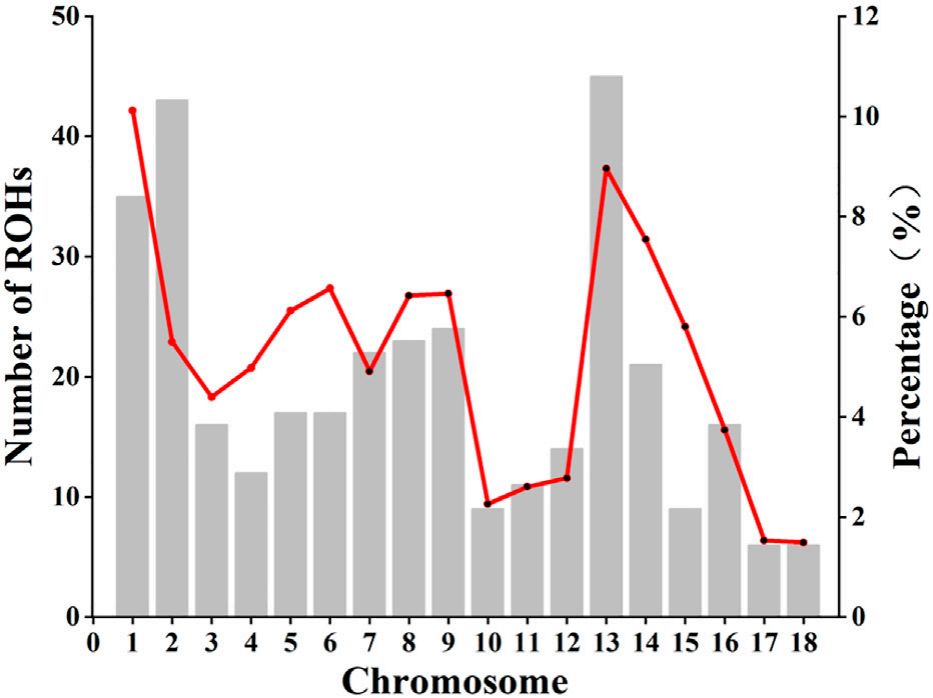
The number of runs of homozygosity (ROH) on each chromosome and the proportion covered by ROH. Bars and the red line represent the number of ROH and the ROH coverage, respectively, on each chromosome.

A total of 184 ROHs was detected from the entire genome with an average ROH length of 23.71 Mb, ranging from 11.26 Mb to 69.02 Mb. The average number of ROH per individual ranged from 1 to 9, and the total length of ROH per individual was mainly distributed between 50 Mb and 75 Mb. The ROH number and the coverage ROH on each chromosome were illustrated in Figure 3. The length of ROH per chromosome was decrease with the decreased chromosome length (Supplementary Figure S3). The number of ROHs was large on chromosome 1, 2, 3, and 13, while the smallest number of ROHs was observed on chromosome 17 and 18 (Supplementary Table S2). The length of ROH was largest on chromosome 1 with an average ROH coverage of 10.12%, whereas the lowest ROH number was observed on chromosome 18 (ROH coverage of 1.49%). We excluded sibling effects on F_ROH_ and calculated F_ROH_ for 41 individuals without full-sib families. Based on ROH data, individual F_ROH_ was evaluated as 0.04%, indicating a low genomic inbreeding level.

**FIGURE 4:**
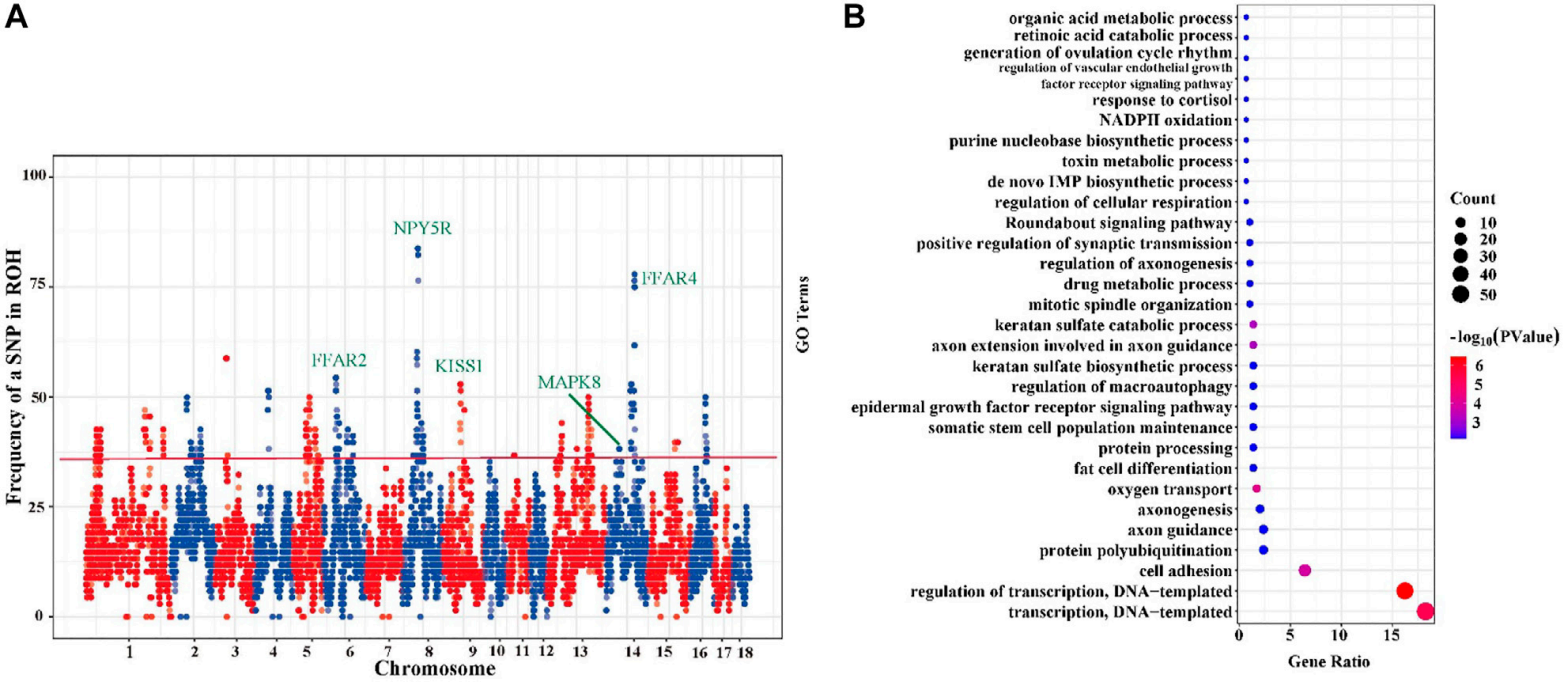
Incidence of each SNP in all runs of homozygosity (ROH) and the GO enrichment analysis of candidate genes. **(A)** Incidence of each SNP in all ROH segment. Particular genomic positions can be seen, on chromosomes 3,4,6,8,9,13,14,15,16 featured in ROH which were shared in over 50% of the sampled animals. **(B)** Bubble chart of GO terms enriched (*p* < 0.05) based on ROH islands. The larger the bubble, the more genes are enriched in this term, and the darker the red, the smaller the *p* value.

As shown in Figure 4A, the percentage of SNPs in ROH region varied with chromosome. The most frequent SNP in ROH region (61 occurrences, 89.71%) was mapped at ∼54 Mb on *sus scrofa* chromosome (SSC) 8. We identified a total of 14 ROH island regions (Supplementary Table S3) with minimum region length of 0.78 Mb on SSC 14 and maximum of 5.43 Mb on SSC 11, and a total of 305 SNPs were detected from these 14 ROH islands. Subsequently, the detected 305 SNPs were genetically annotated, and a total of 240 candidate genes were obtained. All these 240 candidate genes located in ROH islands were subjected to GO enrichment analysis and these genes were found to be significantly enriched in 30 GO terms (*p* < 0.05) (Figure 4B, Supplementary Table S4). Among these terms, we focused on three important terms related to the germplasm characteristics of TC pigs, namely “fat cell differentiation (GO:0045444),” “regulation of macroautophagy (GO:0016241),” “generation of ovulation cycle rhythm (GO:0060112)”. TC pigs as germplasm resources are characterized by excellent meat quality, moderate intramuscular fat, and strong flavor. We identified several important candidate genes that may be involved in the formation of these genetic characteristics, such as *FFAR2*, *FFAR4*, *MAPK8*, *NPY5R*, and *KISS1*.

## Discussion

The alleles of TC pigs were expected to be diverse due to the absence of intense directional selection of production traits. Maintaining genetic diversity within and across breeds is crucial for sustainable development of livestock production (Ollivier and Foulley, 2005). To reveal genetic structure and diversity of TC pigs, we investigated population structure, linkage disequilibrium, effective population size, genetic diversity and runs of homozygosity based on genome-wide SNP data.

### Population structure of TC pigs

To understand the population structure, we conducted MDS analysis and constructed Neighbor-joining (NJ) tree. Figure 1A showed a weak differentiation between 68 TC pigs, which indicated that TC breeding pigs conservation population is an unstratified purebred population. Furthermore, NJ tree showed a sufficient pedigree number (10), and the number of boars in each pedigree varied from 1 to 3, suggesting that there was an uneven distribution of families in this population. The uneven family distribution will increase the possibility of decreasing genetic diversity of TC pigs. To avoid the reduction in genetic diversity and inbreeding depression, it is recommended to maintain equivalent numbers of boar in each family.

### Genetic diversity of TC pigs

Genetic diversity is affected by various factors. This study comprehensively assessed the genetic diversity of TC pigs by investigating LD, Ne, genetic diversity parameters (P_N_, MAF, heterozygosity, and Pi) and neutrality tests. All these parameters indicated a significant level of genetic diversity.

LD describes the non-random association of alleles at different loci, and this association results from processes such as migration, selection, and genetic drift in finite populations (Wang, 2005). The pattern of LD decay can provide information on the evolution of a population. Therefore, comparing the extent of LD between populations can reveal the overall diversity level of various populations. As expected, the most tightly-linked SNP pairs in TC pig genome exhibited the highest r^2^, and average r^2^ was rapidly decreased with the increasing distance between pairwise SNPs, which was consistent with the pattern observed in previous studies of pigs and in other species (Ai et al., 2013; Chen J. et al., 2018). The distance between pairwise SNPs in TC pigs was 15 kb when r^2^ = 0.3 (Jungerius et al., 2005). Previous studies of the genetic diversity have shown that commercial pigs generally have greater LD than Chinese indigenous pigs, with physical distance between pairwise SNPs ranging from 334 kb (Landrace) to 757 kb (White Duroc) for commercial pigs and from 4.5 kb (Wuzhishan) to 744 kb (Min) for Chinese pigs, and 1.5 kb for wild pigs (Ai et al., 2013; Wang et al., 2018; Xu et al., 2019; Liu et al., 2020; Wang et al., 2021). In this study, the LD extension of TC pigs was much smaller than commercial pigs, and it was at low level among Chinese indigenous pigs. LD can reflect the genomic diversity and haplotype diversity of the population. Genome diversity has been reported to be decreased with the LD extension increasing (McVicker et al., 2009). Based on this, it could be speculated that the genetic diversity of the TC pigs is more likely to remain stable. In addition, the sharp decline of LD within short distance (15 kb) indicated high haplotype diversity in the studied population (Figure 2A), and the similar trend has been reported in cattle (Shin et al., 2013; Kim et al., 2018). When a new mutation occurs in a finite population, LD is affected, and the influence degree is dependent on the frequency of the allele induced by the new mutation. With the accumulation of copies of the mutant allele, the LD between this mutated allele and other alleles depends on recombinant rate, random drift, population admixture, and selection. The LD between mutated alleles and their neighboring alleles is generally lower than the average LD. In this study, more new alleles were generated in recent generations of TC pigs with no artificial selection, which might be one of reasons for the rapid decay of LD. This result indicated the abundant genetic variation of TC pig genome.

Linkage disequilibrium (LD) can provide an insight into the evolution of one population. The degree of LD between SNPs can be used to infer ancestral Ne, and Ne is the number of individuals of an ideal population with the same inbreeding coefficients as the actual breeding population (Frankham, 1996). Balanced polymorphisms will be lost when population size becomes too small (Kimura, 1964), thus reducing the genetic diversity of populations. A large effective population size contributes to the conservation of numerous genetic variations. Our data indicated that the Ne of TC before generation 12 (Ne_12_) was 64, which was larger than the minimum threshold (50) set by the Food and Agricultural Organization (FAO, 2000), and that the average Ne before generation 20 (Ne_20_) was estimated to be 96. The Ne_20_ of TC pigs was generally smaller than that of global pig breeds such as the Large White (214.4), Duroc (207.2) and Landrace (207.5) (Traspov et al., 2016), and it was comparable to Chinese indigenous pig breeds, which ranged from 85.0 for Shaziling pigs to 165.0 for Erhualian pigs (Wang et al., 2018). This result might be explained by the fact that the population of the Chinese indigenous breeds were smaller than global pig breeds due to the extensive importation of commercial breeds into China during past decades. A comprehensive population structure analysis showed that TC pig families with fewer boars needed to increase their numbers. Further, it is possible to properly introduce purebred TC pigs from other protected areas and increase the pedigree of TC pigs, which can effectively increase the population size.

It has been reported that Chinese indigenous pigs are richer in genetic variability than commercial pigs (Ai et al., 2013; Wang et al., 2015). Hence, Chinese indigenous pigs are expected to have larger Ho. Our estimated heterozygosity values (average Ho 0.32, and He 0.31) were consistent with those in most previous studies of different pig populations (Ai et al., 2013; Traspov et al., 2016; Wang et al., 2018). Our Ho values were considerably higher than those reported in previous studies of cosmopolitan pig breeds, which ranged from 0.25 (Hampshire) to 0.34 (Large White) (Traspov et al., 2016), and our Ho values of TC breed were similar to those of some Chinese local breeds ranging from 0.15 of Meishan breed to 0.349 of Laiwu breed (Traspov et al., 2016; Wang et al., 2018; Xu et al., 2019; Liu et al., 2020; Wang et al., 2021). Our He values of TC breed were similar to those of cosmopolitan pig breeds, which ranged from 0.27 (Berkshire) to 0.395 (Landrace) (Wang et al., 2018), but they were higher than those of some Chinese local breeds ranging from 0.14 (Jinhua) to 0.4 (Kele) (Ai et al., 2013; Xu et al., 2019). In addition to the heterozygosity, MAF is also extensively used for genetic diversity studies since it can distinguish between common and rare variant in the population, and A higher proportion of low MAF values is associated with high genetic diversity is associated with high genetic diversity (Murray et al., 2010; O'Brien et al., 2014). In this study, the mean MAF was 0.23, and the proportion of MAF<0.1 was high, which indicated the high genetic diversity of the TC pig population. Pi are the basic parameters to assess genetic diversity. Pi is to measure the degree of intrapopulation haplotype mutation. Our results showed that the TC pig population had higher nucleotide diversity (Pi, 0.31) than Chinese indigenous pig breed Bamei (Pi, 0.01) and commercial breed (Pi, 0.01–0.02) (Zhang et al., 2018). The high Pi of the genome provided support for the fast-decaying LD. Additionally, the positive mean values of Tajima’s D, Fu and Li’s F*, and Fu and Li’s D* TC indicated that pigs might have experienced recent size reductions, but they might not indicate the deviations from the neutral expectation. The studies of molecular and phenotypic evolution have revealed that positive selection of advantageous mutations drives divergence between populations (species) and reduce polymorphism within populations (species) (Suzuki, 2010). All our parameters showed a high level of genetic diversity of TC pigs. Since genetic diversity is affected by multiple factors, genetic variation should be continuously monitored in breed conservation to prevent an irreversible erosion of genetic diversity of livestock populations, thus maximizing breed adaptability (Biscarini et al., 2015).

### ROH distribution and candidate genes of tongcheng pigs

#### ROH level of TC pigs

Short ROH segments are known as indicators of distant consanguinity, whereas long ROH segments are more likely to result from recent inbreeding (Curik et al., 2014). Average ROH length of Chinese pigs varied considerably, ranging from the lowest value of 20.6 Mb in wild boar to the largest value of 168 Mb in Diannanxiaoer (Wang et al., 2018).

Broman and Weber (1999) first identified long homozygous segments in the human genome, and Gibson et al. (Broman and Weber, 1999) described the potential of long homozygous segments for inbreeding assessment. F_ROH_ was first defined by McQuillan et al. (2008). Since 2010, F_ROH_ is an accurate estimator of inbreeding coefficient, and thus it is used for studying inbreeding and selection in animal populations. F_ROH_ was the first reported in 2012 in pigs (Bosse et al., 2012), and recently, it has been used for calculating inbreeding coefficient of Jinhua pigs (F_ROH_ 0.17) (Xu et al., 2019). For example, the Laiwu pig breed has shrunk with the introduction of a large number of commercial breeds, and its F_ROH_ was 0.06 in 2021 (Fang et al., 2021). In this study, we excluded sibling effects on F_ROH_ and calculated F_ROH_ for 41 individuals without full-sib families. TC pigs exhibited a low F_ROH_ (0.04%), indicating a low inbreeding level and preservation of genetic diversity.

#### ROH islands and candidate genes

ROH contributes to the investigation of inbred genomic regions within a population, which were first defined as ROH islands by Nothnagel et al. (2010). Selection leaves certain peaks across the genome, and these peaks are called hotspots and considered to be the signal of selective sweeps (Curik et al., 2014). These hotspots (stretches of homozygous sequences) are shared by a large proportion of individuals in a population, and they are designated as ROH islands.

For instance, some candidate genes such as *FFAR2*, *FFAR4*, *MAPK8*, *NPY5R*, and *KISS1* located in ROH islands were found to be related to fat cell differentiation, fat deposition, and reproduction. Of them, *FFAR*2 and *FFAR4* (free fatty acid receptors 2 and 4) were mammalian receptor of short-chain fatty acids (SCFAs). Free fatty acid receptors (*FFAR*) were found on the apical or basolateral membranes of enteroendocrine and immune cells at the gut mucosal lining (Rohrl et al., 2011; Mielenz, 2017; Psichas et al., 2017; Kimura et al., 2020). Previous study has reported that the expression of *FFAR*2 is potentially related to the abundance of short chain fatty acids (Vigors et al., 2019). Recent research has indicated that SCFAs might affect immunity, metabolism, and the pathogenesis of obesity, diabetes, and inflammatory bowel disease in humans through gut microbiota (Meijer et al., 2010). One study of knockout mice has indicated that *FFAR*2 plays a major role in mediating the effect of gut microbiota on host obesity and immunity (Bjursell et al., 2011). However, studies in pigs have found that the adipogenic effect of SCFAs in porcine is unlikely to be mediated by *FFAR*2 (Li et al., 2014). TC pigs have excellent meat quality and flavor, indicating that gene *FFAR* may play a key role in fatty acid composition in TC pig population, but more evidence is needed. Candidate gene *MAPK8*, harboured on ROH islands, has been reported to control an important trait in TC pigs, and this gene is significantly correlated with backfat thickness in carcasses (Otieno et al., 2005). In addition, the mitogen-activated protein kinase (*MAPK*) signaling pathway participates in multiple biological processes including innate immunity, cell growth, stress response, apoptosis, and differentiation (Purves et al., 2001). The *MAPK* signaling pathway is activated by cells after infection and intoxication (Widmann et al., 1999). Previous study has shown that the TC pig strong resistance to highly pathogenic PRRSV infection (Liang et al., 2017), indicating that gene *MAPK8* might play a important role in PRRSV infection in TC pig population, but more evidence is needed. Neuropeptide Y5 receptor (*NPY5R*) is expressed in hypothalamic areas that control feeding (Larsen et al., 1993). The *NPY* (including *NPY1R* and *NPY5R*) is mainly responsible for promoting food intake and reducing energy consumption as well as fat deposition in mice (Nguyen et al., 2012). The selection signals of *NPY5R* genes have also been detected in multiple Chinese local pig breeds, such as Tongcheng (Wang et al., 2015), Rongchang, and Jinhua (Elbers et al., 2009), Laiwu (Chen M. H. et al., 2018), and Anqing six-end-white pigs (Zhang et al., 2020). Kisspeptin (first named metastin), a peptide product of *KISS1* gene, is a neuropeptide responsible for regulating reproduction (Ohtaki et al., 2001). Kisspeptin effectively stimulates LH and FSH release in prepubertal gilts (Lents et al., 2018). TC pigs have early sexual maturity, and the sows exhibit sexual maturity at the age of 4–5 months (Data unpublished). Therefore, *KISS1* gene may be an essential gene affecting the reproduction performance of sows, which deserves more attention.

## Conclusion

Population structure and genetic diversity are of great importance for genetic improvement programs of indigenous pig and the conservation and effective use of genetic resources. The present study provides insights into the population structure and genetic diversity of TC pigs in China. The results showed that the studied TC pig populations were free from admixture of other breeds. Our investigated parameters such as P_N_, MAF, He and Ho, and nucleotide diversity showed a remarkably high level of genetic diversity of TC pigs. The population structure and genetic diversity analyses indicated that TC pigs represented a valuable genetic resource, but their breed conservation program remains to be further optimized so as to ensure adequate genetic diversity and avoid inbreeding depression. Our findings lay a theoretical basis for conservation of TC pigs and provide genome data for subsequent studies of meat traits such as fatty acids and meat quality.

## Data Availability

The original contributions presented in the study are included in the article/Supplementary Material, further inquiries can be directed to the corresponding author.
