# Supplementary material for: Genetic diversity and population structure of Tongcheng pigs in China using whole-genome SNP chip
Source: Front Genet. 2022 Aug 25;13:910521. doi: 10.3389/fgene.2022.910521 (PMC9455598; doi:10.3389/fgene.2022.910521)
Supplement: Supplementary file 1 [file DataSheet1.docx]

Supplementary Material

# Supplementary Figures


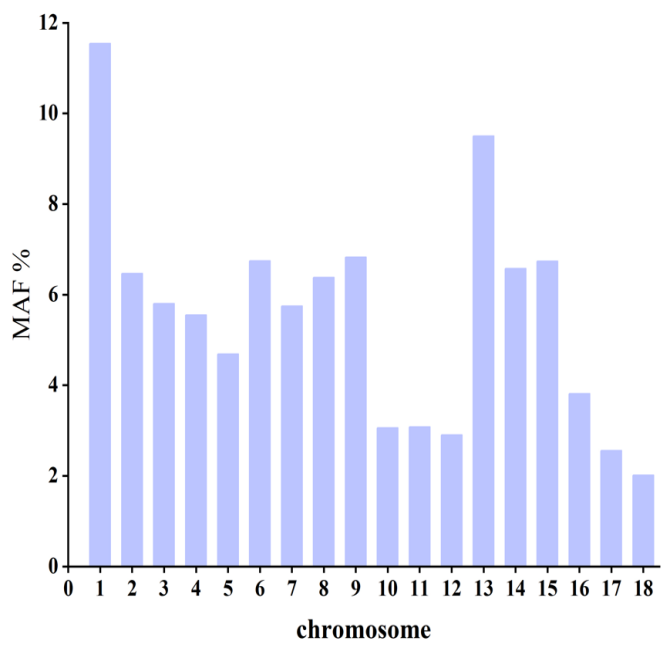

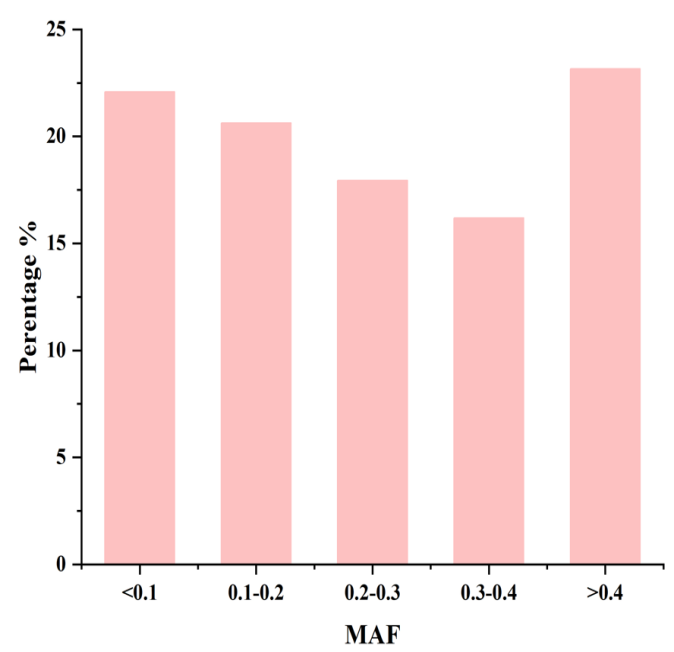


**（A）**

**(B)**

Supplementary Figure 1. **Distribution of the** minor allele frequency （MAF） **of 26999 SNP markers used in current study.**

(A) The percentage of MAF per chromosome. (B) Distribution of the MAF in frequencies <0.1, 0.1~0.2, 0.2~0.3, 0.3~0.4, and >0.4.


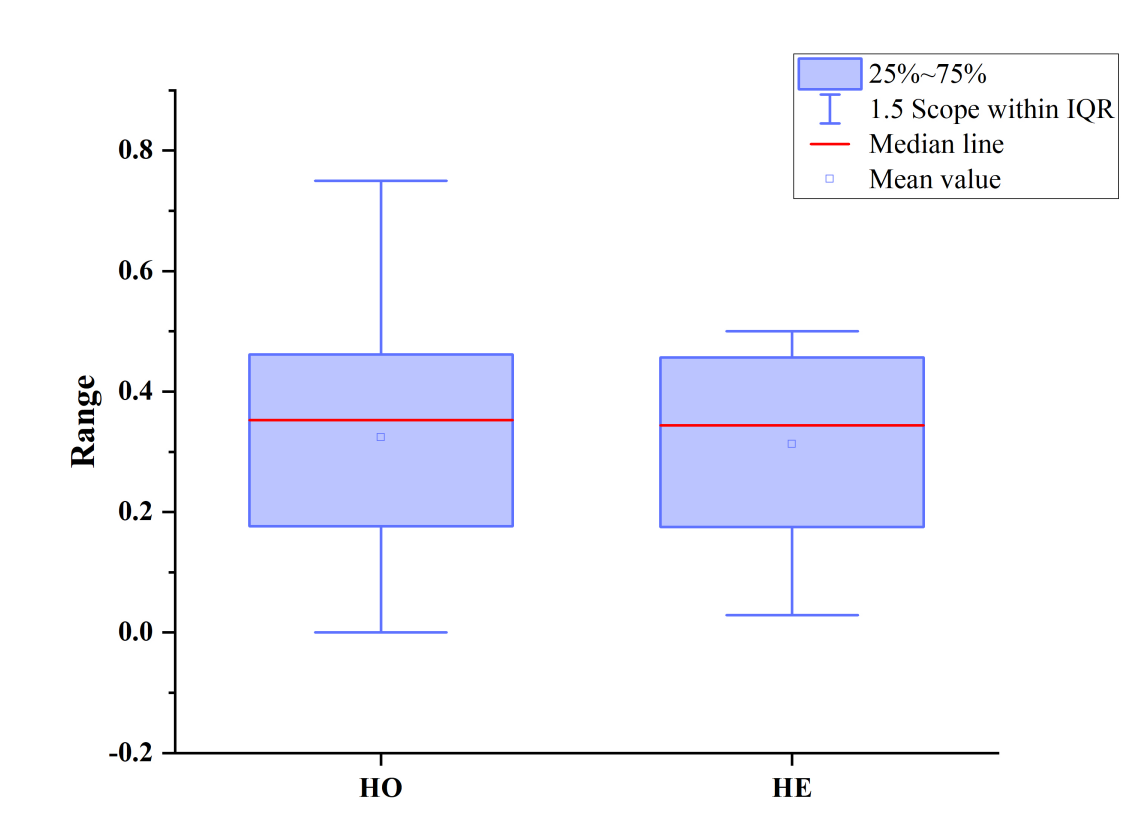


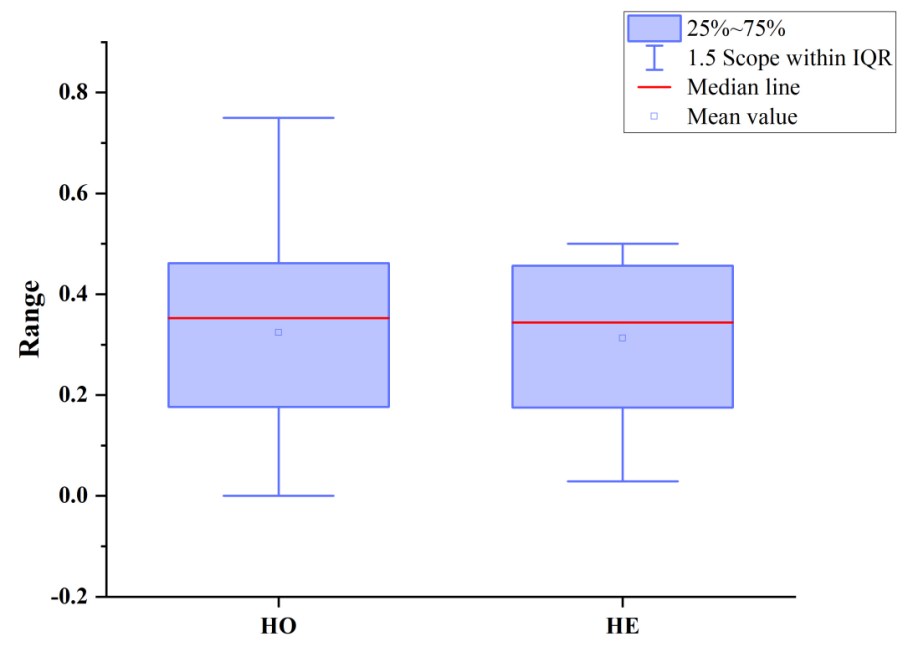

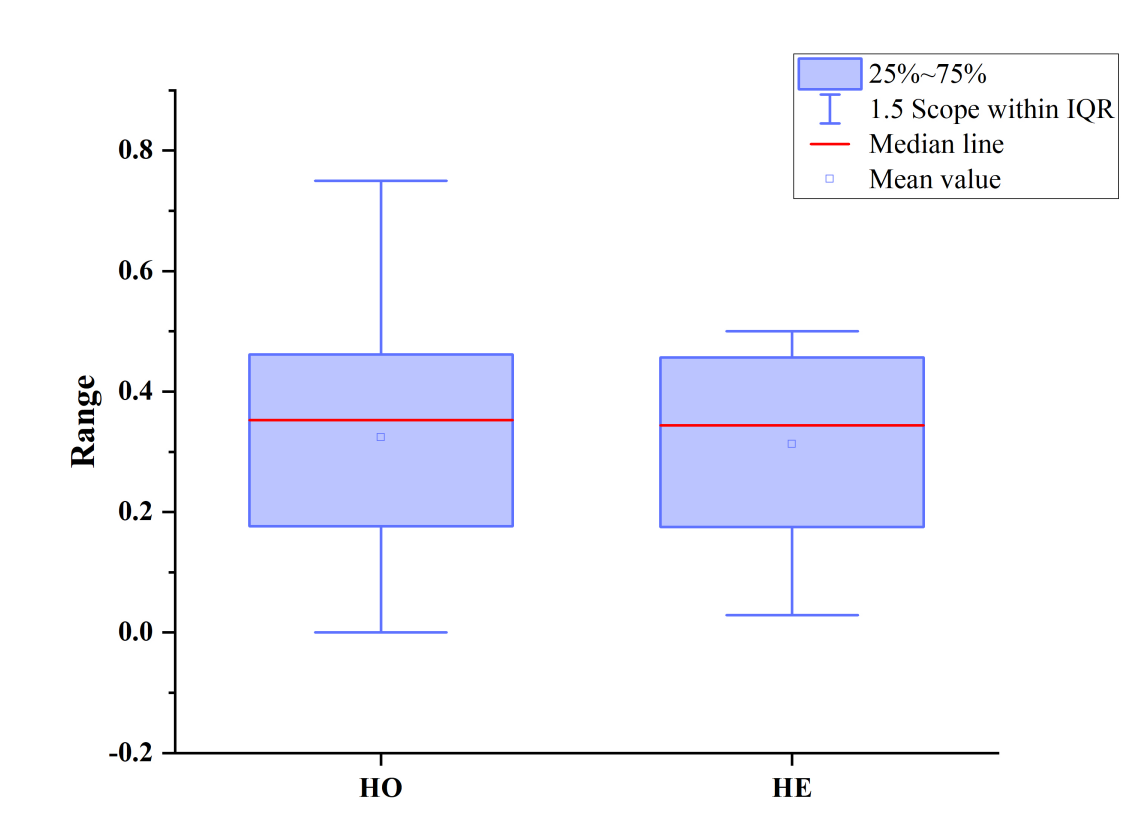


- Supplementary Figure 2. **Boxplot with observed heterozygosity (HO) and expected heterozygosity (He) of Tongcheng pigs.**The red line represents the median, and the open square represents the mean of Ho (0.32) and He (0.31).


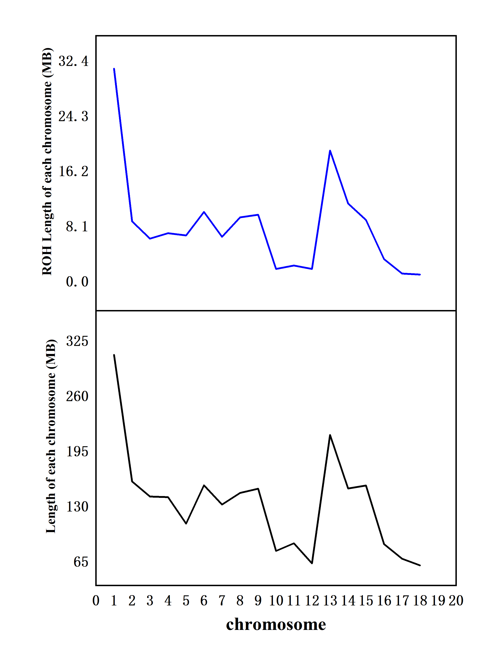


- Supplementary Figure 3. **The total length of ROH per chromosome and the total length of each chromosome distributed in Tongcheng pigs.** The blue line represent the total ROH length distribution per chromosome. The black line represent the total length of per chromosome.

# Supplementary Tables

- Supplementary Table 1. **The percentage of chromosomes covered by ROH of Tongcheng pigs.**

| Chr | Total length of chromosomes(Mb) | Total Length of ROH(Mb) | Percentage (%) |
| --- | --- | --- | --- |
| 1 | 307.91 | 31.15 | 10.12 |
| 2 | 158.53 | 8.71 | 5.49 |
| 3 | 140.80 | 6.19 | 4.40 |
| 4 | 140.10 | 6.98 | 4.98 |
| 5 | 108.89 | 6.66 | 6.12 |
| 6 | 153.98 | 10.11 | 6.56 |
| 7 | 131.41 | 6.46 | 4.91 |
| 8 | 145.00 | 9.31 | 6.42 |
| 9 | 149.99 | 9.69 | 6.46 |
| 10 | 76.77 | 1.73 | 2.26 |
| 11 | 85.61 | 2.23 | 2.60 |
| 12 | 62.10 | 1.72 | 2.77 |
| 13 | 213.50 | 19.12 | 8.96 |
| 14 | 150.23 | 11.33 | 7.54 |
| 15 | 153.95 | 8.93 | 5.80 |
| 16 | 84.84 | 3.17 | 3.74 |
| 17 | 67.62 | 1.04 | 1.53 |
| 18 | 59.78 | 0.89 | 1.49 |

| **Chr** | **Start (bp)** | **End (bp)** | **Length (bp)** | **Number of SNPs** |
| --- | --- | --- | --- | --- |
| 1 | 38,700,672 | 39,675,311 | 974,639 | 12 |
| 1 | 50,453,600 | 52,156,856 | 1,703,256 | 20 |
| 1 | 284,998,024 | 286,549,851 | 1,551,827 | 14 |
| 3 | 39,046,431 | 42,096,113 | 3,049,682 | 22 |
| 5 | 48,459,212 | 49,669,347 | 1,210,135 | 17 |
| 6 | 44,482,860 | 46,554,254 | 2,071,394 | 20 |
| 6 | 57,014,421 | 58,475,039 | 1,460,618 | 20 |
| 8 | 52,075,182 | 53,042,867 | 967,685 | 8 |
| 8 | 54,181,832 | 57,337,293 | 3,155,461 | 33 |
| 9 | 63,397,420 | 65,264,546 | 1,867,126 | 23 |
| 13 | 148,782,749 | 154,341,569 | 5,558,820 | 37 |
| 14 | 89,058,255 | 93,519,479 | 4,461,224 | 35 |
| 14 | 103,312,088 | 105,455,931 | 2,143,843 | 32 |
| 16 | 54,891,348 | 55,691,446 | 800,098 | 8 |

- Supplementary Table 2. **List of genomic regions of ROH island detected in Tongcheng pigs.**
- Supplementary Table 3. **GO enrichment analysis 240 candidate genes located in ROH islands.**

| **Term** | **Count** | **P Value** | **Genes** |
| --- | --- | --- | --- |
| GO:0006355~regulation of transcription, DNA-templated | 48 | 3.77E-07 | ENSG00000198482, ENSG00000185869, ENSG00000197372, ENSG00000175322, ENSG00000197134, ENSG00000127081, ENSG00000188283, ENSG00000134532, ENSG00000153896, ENSG00000196967, ENSG00000085644, ENSG00000197928, ENSG00000197808, ENSG00000185177, ENSG00000127903, ENSG00000165606, ENSG00000183850, ENSG00000084093, ENSG00000256771, ENSG00000105497, ENSG00000131653, ENSG00000197360, ENSG00000180884, ENSG00000197020, ENSG00000114439, ENSG00000130182, ENSG00000198039, ENSG00000196357, ENSG00000189042, ENSG00000196437, ENSG00000118620, ENSG00000188171, ENSG00000146757, ENSG00000245680, ENSG00000196705, ENSG00000143842, ENSG00000189164, ENSG00000171827, ENSG00000128805, ENSG00000010539, ENSG00000122386, ENSG00000103326, ENSG00000121570, ENSG00000161298, ENSG00000237440, ENSG00000254004, ENSG00000167635, ENSG00000140992 |
| GO:0006351~transcription, DNA-templated | 54 | 4.70E-06 | ENSG00000185869, ENSG00000197372, ENSG00000197134, ENSG00000196967, ENSG00000145934, ENSG00000197928, ENSG00000197808, ENSG00000127903, ENSG00000183850, ENSG00000167967, ENSG00000084093, ENSG00000131653, ENSG00000197360, ENSG00000113645, ENSG00000171476, ENSG00000196357, ENSG00000196437, ENSG00000188171, ENSG00000196705, ENSG00000171827, ENSG00000128805, ENSG00000152804, ENSG00000122386, ENSG00000121570, ENSG00000237440, ENSG00000254004, ENSG00000272333, ENSG00000140992, ENSG00000198482, ENSG00000105258, ENSG00000175322, ENSG00000127081, ENSG00000188283, ENSG00000153896, ENSG00000085644, ENSG00000185177, ENSG00000165606, ENSG00000256771, ENSG00000105497, ENSG00000180884, ENSG00000197020, ENSG00000047315, ENSG00000114439, ENSG00000130182, ENSG00000198039, ENSG00000005513, ENSG00000189042, ENSG00000118620, ENSG00000146757, ENSG00000245680, ENSG00000143842, ENSG00000189164, ENSG00000010539, ENSG00000161298 |
| GO:0015671~oxygen transport | 5 | 5.23E-05 | ENSG00000206172, ENSG00000130656, ENSG00000188536, ENSG00000206177, ENSG00000086506 |
| GO:0007155~cell adhesion | 19 | 1.37E-04 | ENSG00000105366, ENSG00000126243, ENSG00000129450, ENSG00000105711, ENSG00000127083, ENSG00000170382, ENSG00000170017, ENSG00000163453, ENSG00000168995, ENSG00000105290, ENSG00000102854, ENSG00000129925, ENSG00000126259, ENSG00000105492, ENSG00000161640, ENSG00000105383, ENSG00000254521, ENSG00000142512, ENSG00000082293 |
| GO:0042340~keratan sulfate catabolic process | 4 | 6.05E-04 | ENSG00000127083, ENSG00000188783, ENSG00000122176, ENSG00000106809 |
| GO:0048846~axon extension involved in axon guidance | 4 | 6.05E-04 | ENSG00000122176, ENSG00000106809, ENSG00000184347, ENSG00000170017 |

**Supplementary Table 3. Continued.**

| **Term** | **Count** | **PValue** | **Genes** |
| --- | --- | --- | --- |
| GO:0018146~keratan sulfate biosynthetic process | 4 | 7.59E-03 | ENSG00000127083, ENSG00000188783, ENSG00000122176, ENSG00000106809 |
| GO:0035385~Roundabout signaling pathway | 3 | 7.59E-03 | ENSG00000122176, ENSG00000106809, ENSG00000184347 |
| GO:0007409~axonogenesis | 6 | 7.59E-03 | ENSG00000126243, ENSG00000127083, ENSG00000188783, ENSG00000170382, ENSG00000122176, ENSG00000106809 |
| GO:0050806~positive regulation of synaptic transmission | 3 | 7.59E-03 | ENSG00000108231, ENSG00000109255, ENSG00000170498 |
| GO:0016241~regulation of macroautophagy | 4 | 7.59E-03 | ENSG00000107643, ENSG00000185883, ENSG00000090989, ENSG00000126247 |
| GO:0007411~axon guidance | 7 | 7.59E-03 | ENSG00000145934, ENSG00000105711, ENSG00000133703, ENSG00000108231, ENSG00000165606, ENSG00000162068, ENSG00000184347 |
| GO:0006082~organic acid metabolic process | 2 | 7.59E-03 | ENSG00000094963, ENSG00000010932 |
| GO:0050770~regulation of axonogenesis | 3 | 7.59E-03 | ENSG00000122176, ENSG00000106809, ENSG00000184347 |
| GO:0007173~epidermal growth factor receptor signaling pathway | 4 | 7.59E-03 | ENSG00000138193, ENSG00000133703, ENSG00000114423, ENSG00000140992 |
| GO:0000209~protein polyubiquitination | 7 | 7.59E-03 | ENSG00000072401, ENSG00000145416, ENSG00000107854, ENSG00000059145, ENSG00000114423, ENSG00000198060, ENSG00000103266 |
| GO:0034653~retinoic acid catabolic process | 2 | 7.59E-03 | ENSG00000095596, ENSG00000187553 |
| GO:0060112~generation of ovulation cycle rhythm | 2 | 7.59E-03 | ENSG00000164129, ENSG00000170498 |
| GO:0030947~regulation of vascular endothelial growth factor receptor signaling pathway | 2 | 7.59E-03 | ENSG00000131634, ENSG00000138119 |
| GO:0051414~response to cortisol | 2 | 7.59E-03 | ENSG00000163453, ENSG00000184347 |
| GO:0017144~drug metabolic process | 3 | 7.59E-03 | ENSG00000094963, ENSG00000076258, ENSG00000010932 |
| GO:0035019~somatic stem cell population maintenance | 4 | 7.59E-03 | ENSG00000047315, ENSG00000105258, ENSG00000130182, ENSG00000121570 |
| GO:0007052~mitotic spindle organization | 3 | 7.59E-03 | ENSG00000075702, ENSG00000138160, ENSG00000181392 |
| GO:0070995~NADPH oxidation | 2 | 7.59E-03 | ENSG00000094963, ENSG00000010932 |
| GO:0009113~purine nucleobase biosynthetic process | 2 | 7.59E-03 | ENSG00000128050, ENSG00000128059 |
| GO:0009404~toxin metabolic process | 2 | 7.59E-03 | ENSG00000094963, ENSG00000010932 |
| GO:0006189~'de novo' IMP biosynthetic process | 2 | 7.59E-03 | ENSG00000128050, ENSG00000128059 |
| GO:0016485~protein processing | 4 | 7.59E-03 | ENSG00000119912, ENSG00000007384, ENSG00000205155, ENSG00000103269 |
| GO:0045444~fat cell differentiation | 4 | 7.59E-03 | ENSG00000126262, ENSG00000186188, ENSG00000105270, ENSG00000005513 |
| GO:0043457~regulation of cellular respiration | 2 | 7.59E-03 | ENSG00000084092, ENSG00000122873 |
